# Telomere associated gene expression as well as TERT protein level and telomerase activity are altered in the ovarian follicles of aged mice

**DOI:** 10.1038/s41598-021-95239-5

**Published:** 2021-07-30

**Authors:** Esra Gozde Kosebent, Saffet Ozturk

**Affiliations:** Department of Histology and Embryology, Akdeniz University School of Medicine, Campus, 07070 Antalya, Turkey

**Keywords:** Cell biology, Senescence

## Abstract

Telomeres cap the ends of eukaryotic chromosomes to maintain genomic stability and integrity during an organism’s lifespan. The length of telomeres inevitably shortens due to DNA replication, genotoxic agents, and biological aging. A limited number of cell types, e.g., stem cells, germline cells, and early embryos can elongate shortened telomeres via the enzymatic action of telomerase, which is composed of telomerase reverse transcriptase (TERT) and telomerase RNA component (*Terc*). Additionally, telomere-associated proteins including telomeric repeat binding factor 1 (TRF1) and 2 (TRF2), as well as protection of telomeres 1a (POT1a), bind to telomeres to maintain their structural integrity and length. During ovarian aging in mammals, telomeres progressively shorten, accompanied by fertility loss; however, the molecular mechanism underlying this attrition during follicle development remains unclear. In this study, the primary, secondary, preantral, and antral follicles were obtained either from 6-week-old adult (n = 19) or 52-week-old aged (n = 12) mice. We revealed that the *Tert*, *Terc*, *Trf1*, *Trf2*, and *Pot1a* gene expression (*P* < 0.001) and TERT protein (*P* < 0.01) levels significantly decreased in certain ovarian follicles of the aged group when compared to those of the adult group. Also, telomerase activity exhibited remarkable changes in the follicles of both groups. Consequently, altered telomere-associated gene expression and reduced TERT protein levels in the follicles of aged mice may be a determinant of telomere shortening during ovarian aging, and infertility appearing in the later decades of reproductive lifespan. Further investigations are required to determine the molecular mechanisms underlying these alterations in the follicles during ovarian aging.

## Introduction

Fertility in women progressively decreases during aging due to genetic defects, enhanced rates of implantation failure and pregnancy loss, and dysfunction in the reproductive organs including ovaries^1,2^. Number of follicles at different development stages and quality of oocytes dramatically reduce during biological aging^3,4^. Over the last decades, women have delayed childbearing because of socioeconomic reasons. There is, therefore, an urgent need to characterize molecular processes involved in ovarian aging that contribute to fertility loss. One of the main factors associated with ovarian aging accompanied by infertility is the shortening of telomeres in the oocytes and granulosa cells^5,6^.

Telomeres are non-coding repetitive DNA sequences (TTAGGG)_n_ located at the ends of eukaryotic chromosomes^7^. They primarily attach the ends of chromosomes to the nuclear membrane and nuclear matrix to prevent chromosomal fusion and erroneous DNA recombination events^8^. Additionally, these unique formations are involved in the synapse formation, correct distribution and pairing of chromosomes during mitotic and meiotic divisions. The 3’ end of telomeres composed of G-rich 50–350 nucleotides is known as the G-tail or G-overhang^9^. To preclude any potential nuclease attack, the G-tail folds back to form D- and T-loop structures^5^, which also control elongation of telomeres by regulating the entrance of the enzyme telomerase into telomeric ends. It should be noted that telomeres consist of both DNA sequences and telomere-associated proteins to make up this nucleoprotein complex. The telomere-associated proteins including telomere repeat binding factor 1 (TRF1) and 2 (TRF2), protection of telomeres 1a (POT1a), POT1 and TIN2 interacting protein 1 (TPP1), TRF1 interacting nuclear protein 2 (TIN2), and repressor activator protein 1 (RAP1) are exclusively defined as the shelterin complex, which maintains the integrity of chromosome ends and regulates telomere elongation process^8,10^.

Among these telomere-associated proteins, the TRF1 and TRF2 proteins specifically bind to the double-stranded part of telomeric sequences in order to maintain their length^11,12^. As TRF1 plays a key role in repressing telomerase activity, its overproduction excessively shortens telomeres^13^. It also contributes to DNA replication process by inducing several helicases such as Bloom syndrome helicase (BLM)^14^. Moreover, TRF1 involves in the adhesion of telomeres to the nuclear membrane so that chromosomal fusion is prevented during meiosis in mice^15^. Similarly, TRF2 negatively regulates telomere lengthening^16^, and further functions in prevention of DNA damage response activation to telomeres by establishing an interaction with the ataxia telangiectasia, mutated (ATM) protein kinase^17,18^. Therefore, chromosomal defects, telomere attrition, and enhanced cell death occur in the absence or overproduction of TRF2^16^. Unlike TRF1 and TRF2, the POT1 protein interacts with the single-stranded part of telomeres and contributes to blocking DNA damage response activation to telomeres by repressing the ataxia telangiectasia and Rad3 related (ATR) protein kinase^19^. As POT1 influences telomere length by positively modulating telomerase activity, its absence leads to certain defects related to chromosome pairing and genomic integrity, as a consequence of excessive telomere shortening^20^.

As is known, telomeres shorten due to sequential DNA replication, oxidative stress, genotoxic agents, occurrence of various diseases, and biological aging. Once their length becomes critically short, cellular senescence^21^ and reproductive aging along with increased apoptosis rate may occur^5,22^. Depending on the cell type, short telomeres can be extended by telomerase or via the alternative lengthening of telomeres (ALT) mechanism. Only cell types such as germline, granulosa, stem, endothelial, cancerous, and lymphocytes as well as early embryos exhibit telomerase activity in humans^11,12^. Basically, telomerase is a ribonucleoprotein consisting of two subunits: telomerase reverse transcriptase (TERT) and telomerase RNA component (*Terc*). The catalytic subunit, TERT, possesses reverse transcriptase activity and utilizes a short sequence of *Terc* RNA as a template to synthesize telomeric repeats onto chromosome ends^23^.

The telomere-associated proteins and telomerase components play key roles via regulating telomere length and telomerase activity in the ovarian follicles for successful folliculogenesis^12,24^. As is known, ovarian follicles are basically composed of oocytes arrested at the first meiotic division and surrounding granulosa cells from primordial to antral stages^25^. It is worth noting that a theca layer originating from stromal cells further encloses the granulosa cells from the secondary follicle stage^26^. A limited number of studies analyzed the telomerase activity and telomere length in the mammalian ovarian follicles. In rats, telomerase activity was at higher levels in the small and healthy follicles when compared to the large and atretic follicles^27^. Similarly, telomerase activity gradually decreased from preantral follicles to antral follicles in bovines^28^. In contrast, the length of telomeres was found to be longer in the preantral and antral follicles than in the early follicular stages in pigs^29^. Taken together, while telomerase activity decreases gradually from primordial to antral stages, the length of telomeres increases in the same follicular development direction^11^.

During ovarian aging, the quality of oocytes and the antral follicle count progressively decrease, accompanied by fertility loss^1^. At the same time, the significant changes, e.g., decreasing numbers, regressing structural features, and altered intracellular contents can occur in the oocytes and granulosa cells of the follicles from primary to antral stages due to changes in the reproductive hormones and other physiological factors during aging^30,31^. In mice, it was reported that the telomeres in the follicles at different stages gradually shorten alongside ovarian aging^32^. To the best of our knowledge, the molecular mechanism underlying this shortening process remains unknown. In the present study, we aimed to evaluate the *Tert*, *Terc*, *Trf1*, *Trf2*, and *Pot1a* gene expression in the ovarian follicles (from primary to antral stages) collected from adult and aged mice. The levels of TERT protein and telomerase activity in these follicles were also analyzed.

## Materials and methods

### Animals and sample collection

All the mice used in the present study were housed with free access to food and water under a 12-h light–dark cycle in the Akdeniz University Laboratory Animals Application and Research Centre*.* All experimental protocols were carried out in accordance with relevant guidelines and regulations approved by the Akdeniz University Institutional Animal Care and Use Committee (Protocol number 2017.05.006). Also, all methods were performed in accordance with the ARRIVE guidelines (http://www.nc3rs.org.uk/page.asp?id=1357).

Here, we obtained the ovaries of 6-week-old adult (n = 19) and 52-week-old aged (n = 12) Balb/C female mice. These ovaries were transferred into a petri dish including Dulbecco's Modified Eagle Medium/Nutrient Mixture F-12 (DMEM:F12) media (catalog number 11330–032, Thermo Fisher Scientific, Paisley, UK) and punctured using a 23-gauge-needle under a dissecting microscope (Zeiss, Oberkochen, Germany). We collected ovarian follicles that ranged from primary to antral stages using a mouth-controlled pipette under the dissecting microscope after mechanically removed any stromal cells that surrounded the follicles using the 23-gauge-needle. The follicles were further defined according to the following criteria using an inverted microscope (Diaphot 300, Nikon, Tokyo, Japan). Primary follicles were distinguished by visible and central oocytes surrounded by a layer of cuboidal granulosa cells; secondary follicles possessed two or more layers of granulosa cells enclosing oocytes. We identified preantral follicles by the presence of small spaces among the granulosa cells surrounding centrally located oocytes. Antral follicles were characterized by a single large antrum and eccentrically located oocytes surrounded by specialized granulosa cells known as cumulus cells. The isolated intact follicles from primary to antral stages either from adult or aged mice were subsequently used in the analyses described below.

### Gene expression analysis

Using RNAqueous-Micro Kit (Ambion Inc., Austin, Texas, USA), total RNA was extracted from primary (n = 32 and 25), secondary (n = 45 and 50), preantral (n = 35 and 36) and antral (n = 31 and 24) follicles from 14 adult and 9 aged mice, respectively. The concentration and absorbance values of the isolated RNA were measured by using the Epoch Microplate (BioTek Instruments, Inc., Winooski, Vermont, USA). Any potential genomic DNA contamination was eliminated via addition of DNase (Ambion Inc.) to the extracted RNA. We subsequently synthesized complementary DNA (cDNA) using a RETROscript Reverse Transcription Kit (Ambion Inc.) according to the manufacturer’s instructions.

The relative *Tert*, *Terc*, *Trf1*, *Trf2*, and *Pot1a* gene expression levels were detected by quantitative real-time PCR (qRT-PCR). Each 25 μL reaction volume was composed of 12.5 μL of SYBR Green Supermix (Qiagen Inc., Valencia, CA, USA), 10 μM of primers and 1 μL of cDNA. PCR cycling was carried out in a Rotor-Gene instrument (Corbett Research, Sydney, Australia). The primer sequences used are presented in Table 1. qRT-PCR was assayed in triplicate for each group, meaning three technical repeats. Furthermore, specificity of the PCR products was confirmed using the melting curve analysis. The *β-Actin* gene expression was used as an internal control to normalize the relative expression levels of the target genes. Fold changes were calculated using the 2^−ΔΔCt^ (where Ct stands for cycle threshold) formula.

**Table 1:** The primer sequences and product sizes of the genes.

Gene	Primer (5′–3′)	Product size (bp)
*β-Actin*	P_F_: TGCGTGACATCAAAGAGAAG P_R_: CGGATGTCAACGTCACACTT	244
*Tert*	P_F_: CTCTCTGCTGCGCAGCCGATAC P_R_: CCTCGTTAAGCAGCTCAAAG	287
*Terc*	P_F_: CATTAGCTGTGGGTTCTGGTCT P_R_: TCCTGCGCTGACGTTTGTTT	134
*Trf1*	P_F_: TCTAAGGATAGGCCAGATG CCA P_R_: CTGAAATCTGATGGAGCACGTC	185
*Trf2*	P_F_: TCAGCTGCTTCAAGTACAATGAG P_R_: GGTTCTGAGGCTGTCTGCTT	91
*Pot1a*	P_F_: TCTTCGGTTGTGGAAAGCCT P_R_: TGTTTGATGAAAAATCCTCTCACAG	170

*β-Actin* beta actin, *Tert* telomerase reverse transcriptase, *Terc* telomerase RNA component, *Trf1* telomeric repeat binding factor 1, *Trf2* telomeric repeat binding factor 2, *Pot1a* protection of telomeres 1a genes used in this study, *P*_*F*_ forward primer, *P*_*R*_ reverse primer, *bp* base pair.

### Analysis of TERT protein levels

The TERT protein levels in the primary (n = 60 and 46), secondary (n = 77 and 58), preantral (n = 59 and 64), and antral (n = 56 and 38) follicles isolated from 14 adult and 9 aged mice, respectively were analyzed using western blot (WB) method. The isolated and pooled follicles at each stage were immersed in the lysis buffer [composed of 1% sodium dodecyl sulfate (SDS), 1.0 mM sodium ortho-vanadate, and 10 mM Tris; pH 7.4], incubated on ice for 10 min and then centrifuged at 15,000 rpm, at + 4 °C for 10 min. After boiling the samples in 4 × Laemmli buffer (Sigma-Aldrich, St. Louis, MO, USA), they were run on a 10% SDS–polyacrylamide gel electrophoresis (SDS-PAGE) to separate the proteins based on their molecular weight. These proteins were transferred to a PVDF membrane (Roche, Indianapolis, IN, USA) and incubated with anti-TERT antibody [specific to the amino acids sequence ranging 650–700 (locating inside the reverse transcriptase domain of human TERT protein) out of total 1132 amino acids sequence; catalog number bs-0233R, Bioss, Massachusetts, USA; diluted 1:2000 in 5% (w/v) non-fat dry milk in 1 × TBST] overnight at + 4 °C on a rotary shaker.

The membrane was washed with TBST, blocked with 5% non-fat dry milk, and treated with horse radish peroxidase-labeled goat anti-rabbit IgG for 60 min at room temperature. Then, it was washed with TBST again and incubated with ECL Western Blotting Substrate (Pierce, Rockford, IL, USA) for 3 min. After transferring the membrane to a film cassette exposed here for 1 min, the TERT signals were observed following treatment with the developer and fixative solutions. This experimental procedure was technically repeated twice. We measured the bands on the film using the ImageJ software [National Institutes of Health (NIH), Bethesda, Maryland, USA]. The measured TERT protein levels of each follicle stage were normalized to their own glyceraldehyde-3-phosphate dehydrogenase (GAPDH) protein level. In this way, the relative TERT protein levels in the follicles from primary to antral stages in the adult and aged groups were determined.

### Telomerase activity assay

Telomerase activity in the follicles from primary to antral stages was evaluated using the telomeric repeat amplification protocol (TRAP) assay (TRAPeze Telomerase Detection Kit, catalog number S7700, Millipore, Burlington, Massachusetts, USA). For this analysis, we used the primary (n = 34 and 21), secondary (n = 30 and 46), preantral (n = 30 and 25), and antral (n = 30 and 25) follicles from five adult and three aged mice, respectively. The pooled follicles for each developmental stage were lysed in 1 × CHAPS lysis buffer for 30 min on ice. The TRAP assay was performed according to the manufacturer’s instructions. In this assay, we used 7.5 µl of the extract from primary or secondary follicles, and 5 µl from preantral or antral follicles produced from adult and aged groups. The PCR products were separated on 10% polyacrylamide gel at 150 V for 1.5 h. Finally, the gel was stained in ethidium bromide solution [catalog number E1510, Sigma-Aldrich; the stock solution (10 mg/mL) diluted 1:10,000 in deionized water] for 30 min at room temperature in the dark. Then, we washed the gel, and TRAP assay bands were captured using a ChemiDoc MP system (Bio-Rad Hercules, California, USA). The internal control (IC) bands served as a control for amplification efficiency in each reaction and absence of PCR inhibitors. It is important to note that this assay was technically repeated twice.

### Statistical analysis

The data obtained in this study were analyzed by using student’s *t*-test or one-way ANOVA with a suitable post hoc test for determining the statistical significance (Jandel Scientific, SigmaStat for Windows, version 3.0). *P* < 0.05 was considered statistically significant.

## Results

**Figure 1:**
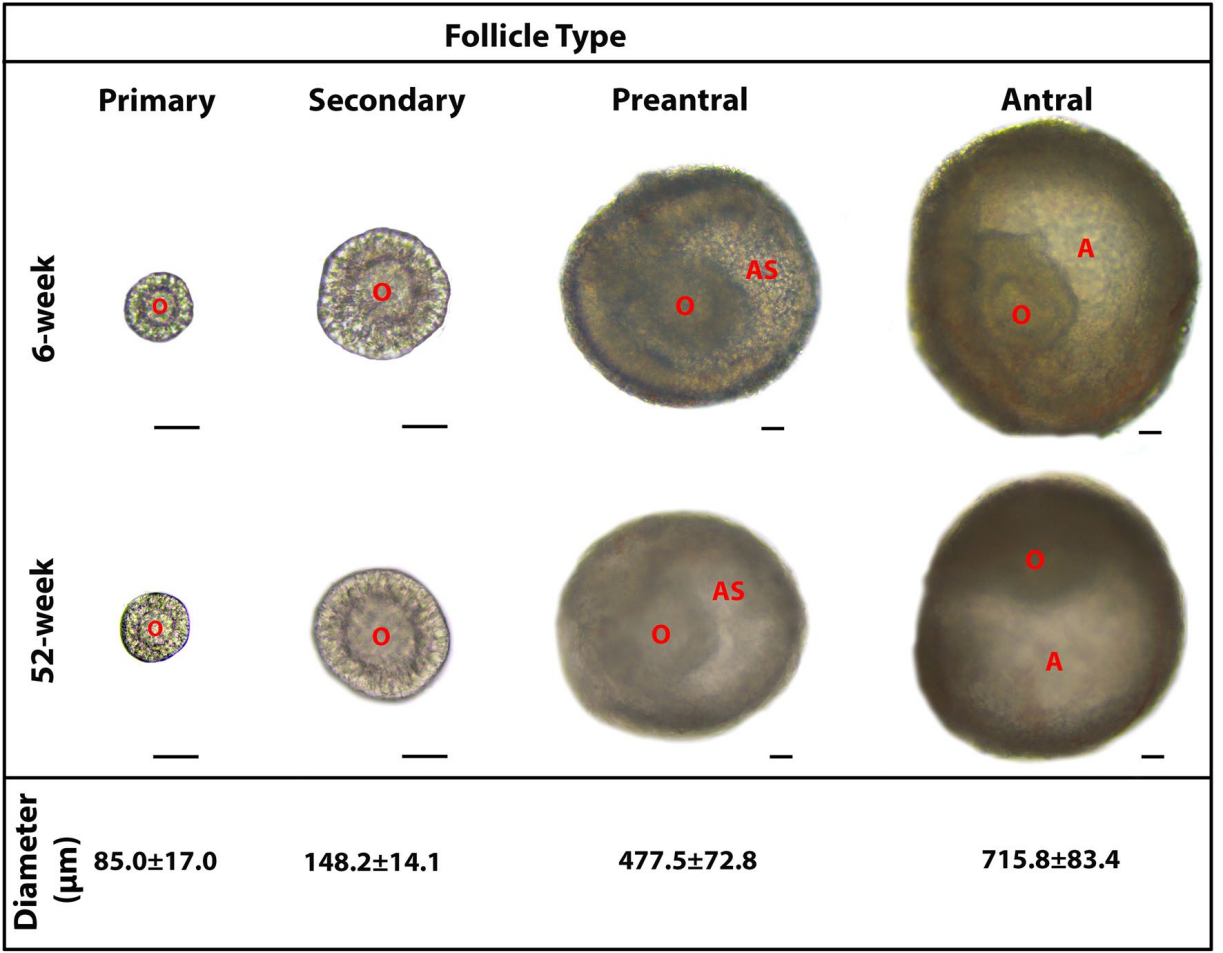
The representative micrographs of the follicles from primary to antral stages, obtained from adult (6-week-old) and aged (52-week-old) mouse ovaries. The micrographs of the follicles were captured under a light microscope (Zeiss, Oberkochen, Germany). All the follicles displayed their characteristic features either in the adult or aged groups. Morphologically normal follicles at similar diameters were used in the subsequent experiments. The average diameter of each follicle type is provided below its own micrograph as mean ± standard deviation (SD). It should be noted that the micrographs of the primary and secondary follicles were captured under an original magnification of 200 ×, whereas those of the preantral and antral follicles were captured at an original magnification of 100 ×. Scale bars are equal to 50 µm. *A* antrum, *AS* antral space, *O* oocyte.

In the present study, we obtained normal appearing and intact follicles at their primary to antral stages either from groups of adult or aged mice. The mean diameters of the follicles from adult and aged groups were measured as 85.0 ± 17.0 µm in the primary follicles, 148.2 ± 14.1 µm in the secondary follicles, 477.5 ± 72.8 µm in the preantral follicles, and 715.8 ± 83.4 µm in the antral follicles (Fig. 1). Follicles with similar diameters were used in the subsequent analyses.

### The *Tert*, *Terc*, *Trf1*, *Trf2*, and *Pot1a* gene expression in the follicles

**Figure 2:**
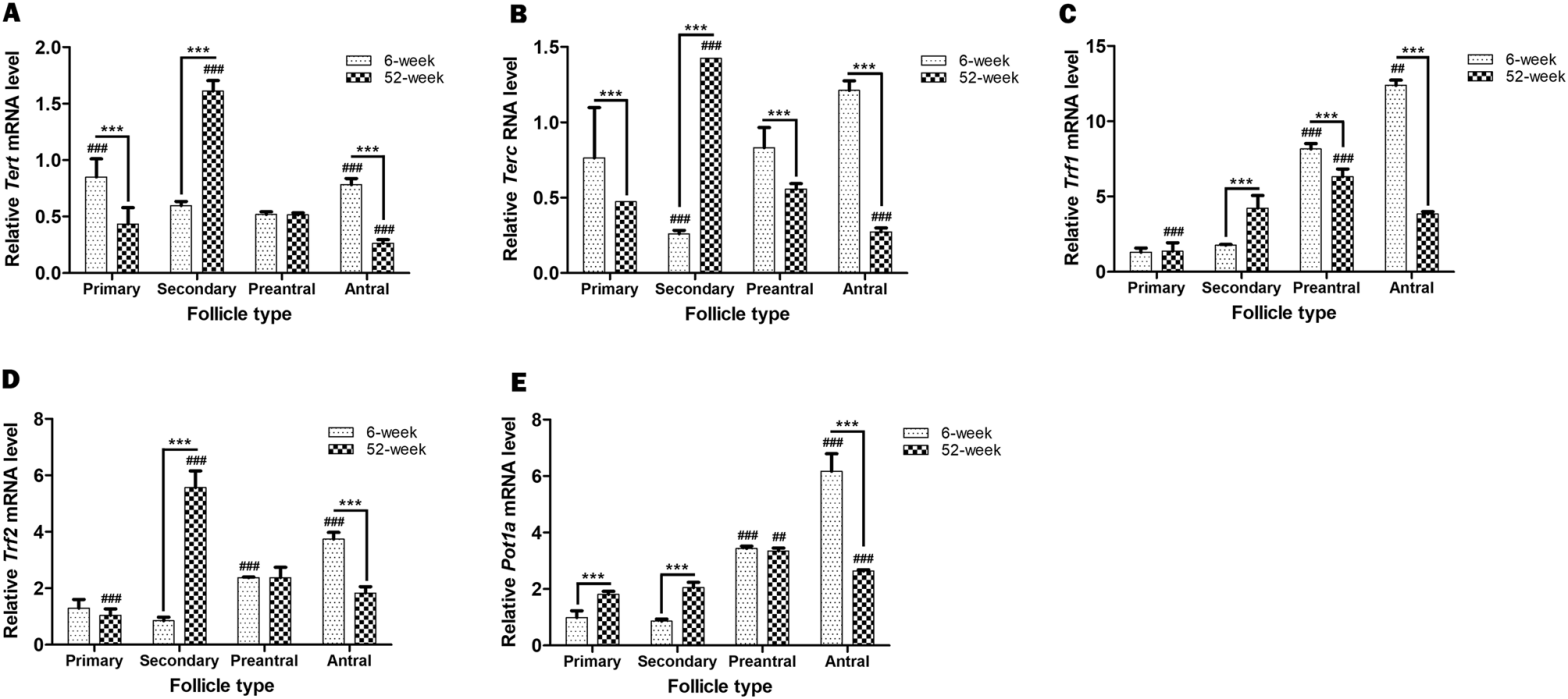
The *Tert*, *Terc*, *Trf1*, *Trf2*, and *Pot1a* gene expressions were evaluated in the ovarian follicles from primary to antral stages, obtained from adult (6-week-old) and aged (52-week-old) groups. Expression levels of these genes were normalized against those of *β-Actin*. It is important to note that expression level of each gene in the primary follicles of the adult group was set to 1 in all analyses. In this analysis, we used primary (n = 32 and 25), secondary (n = 45 and 50), preantral (n = 35 and 36), and antral (n = 31 and 24) follicles from 14 adult and 9 aged mice, respectively. (**A**) The relative *Tert* mRNA levels in the follicles. Although the primary and antral follicles of the aged group displayed lower *Tert* mRNA levels compared to their adult counterparts (*P* < 0.001), the secondary follicles of the aged group exhibited a higher profile than those from adult group (*P* < 0.001). ^###^*P* < 0.001 for the primary follicles versus the secondary and preantral follicles, and the antral follicles versus the preantral follicles in the adult group. ^###^*P* < 0.001 for the secondary follicles versus the remaining follicles in the aged group, as well as for the antral follicles versus the primary and secondary follicles. ***P < 0.001 for the follicles between adult and aged groups. (**B**) The relative *Terc* RNA levels in the follicles. Only the secondary follicles from the aged group exhibited a higher *Terc* RNA level (*P* < 0.001); however, other follicle types displayed lower profiles compared to their adult counterparts (*P* < 0.001). ^###^*P* < 0.001 for the secondary follicles versus the remaining follicles in the adult group. ^###^*P* < 0.001 for the secondary follicles versus the remaining follicles, as well as for the preantral follicles versus the secondary follicles in the aged group. ***P < 0.001 for the follicles between adult and aged groups. (**C**) The relative *Trf1* mRNA levels in the follicles. The secondary follicles from the aged group had significantly higher *Trf1* mRNA levels (*P* < 0.001), whereas the preantral and antral follicles exhibited lower levels, compared to their respective adult counterparts (*P* < 0.001). ^###^*P* < 0.001 for the preantral follicles versus the primary and secondary follicles, ^##^*P* < 0.01 for the antral follicles versus the remaining follicles in the adult group. In the aged group, ^###^*P* < 0.001 for the secondary and antral follicles versus the primary follicles, and for the preantral follicles versus the remaining follicles. ***P < 0.001 for the follicles between adult and aged groups. (**D**) The relative *Trf2* mRNA levels in the follicles. The secondary follicles from the aged group displayed higher *Trf2* mRNA level compared to those of the adult group (*P* < 0.001); this occurrence was reversed in the antral follicles (*P* < 0.001). ^###^*P* < 0.001 for the antral follicles versus the remaining ones, and for the preantral follicles versus the primary and secondary follicles in the adult group. In the aged group, ^###^*P* < 0.001 for the secondary follicles versus the remaining ones, and for the preantral follicles versus the primary follicles. ***P < 0.001 for the follicles between adult and aged groups. (**E**) The relative *Pot1a* mRNA levels in the follicles. The aged group exhibited higher *Pot1a* mRNA levels in the primary and secondary follicles, but lower level in the antral follicles, compared to their adult counterparts (*P* < 0.001). ^###^*P* < 0.001 for the antral follicles versus the remaining ones, and for the preantral follicles versus the primary and secondary follicles in the adult group. In the aged group, ^###^*P* < 0.001 for the antral follicles versus the remaining ones, and ^##^*P* < 0.01 for the preantral follicles versus the primary and secondary follicles. ***P < 0.001 for the follicles between adult and aged groups. Here, the data obtained from the follicles in each group and between the groups were analyzed using one-way ANOVA and the Holm-Sidak post hoc test (denoted by ^##^ or ^###^), and using the student’s *t*-test (denoted by ***), respectively. We presented the values as means ± standard deviations (SDs; arising from three technical repeats).

We first evaluated the *Tert* and *Terc* gene expression levels in the ovarian follicles. In the adult group, detected *Tert* mRNA level in the primary follicles significantly decreased in the secondary and preantral follicles (*P* < 0.001), and subsequently increased in the antral follicles back to level observed in the primary follicles (Fig. 2A; *P* < 0.001). Conversely, the *Tert* mRNA level determined in the primary follicles of the aged group sharply increased in the secondary follicles (*P* < 0.001), then progressively decreased toward antral follicles (*P* < 0.001). We also compared the *Tert* mRNA levels in these follicles between adult and aged groups. The primary and antral follicles in the aged group showed lower levels when compared to their counterparts in the adult group (*P* < 0.001). However, the secondary follicles of the aged group possessed a higher *Tert* mRNA level than those of the adult group (*P* < 0.001); no difference was observed in the preantral follicles between the two groups (Fig. 2A).

The *Terc* RNA level significantly decreased from primary to secondary follicles (*P* < 0.001) and progressively increased in the preantral and antral follicles of the adult group (Fig. 2B; *P* < 0.001). By contrast, its levels in the aged group increased from primary to secondary follicles (*P* < 0.001), then gradually decreased toward antral follicles (*P* < 0.001). By comparing the *Terc* RNA levels among follicles from the adult and aged groups, we observed that the primary, preantral, and antral follicles from the aged group possessed lower levels than those of their adult counterparts (*P* < 0.001). However, the secondary follicles from the aged group had a markedly higher level compared to those from the adult group (Fig. 2B; *P* < 0.001).

The *Trf1* mRNA level gradually increased from primary to antral follicle stages in the adult group (Fig. 2C; *P* < 0.01). In the aged group, it progressively increased from primary to preantral follicles (*P* < 0.001), then remarkably decreased in the antral follicles (*P* < 0.01). A comparison of each follicle type between these two groups revealed that although no difference was observed in the primary follicles, the secondary follicles from the aged group had a higher *Trf1* mRNA level compared to those from adult group (*P* < 0.001). Conversely, its levels in the preantral and antral follicles from the aged group were significantly lower compared to those in the same follicle types from the adult group (Fig. 2C; *P* < 0.001).

The *Trf2* mRNA profile in the adult group showed low levels in the primary and secondary follicles, which progressively increased toward antral follicles (Fig. 2D; *P* < 0.001). In the aged group, the low *Trf2* mRNA level detected in the primary follicles markedly increased in the secondary follicles (*P* < 0.001), then gradually decreased toward antral follicles (*P* < 0.001). Although no differences were detected in the primary and preantral follicles between adult and aged groups, the secondary follicles from the aged group showed higher level when compared to the same follicle type from the adult group (*P* < 0.001). By contrast, the antral follicles from the aged group possessed a lower *Trf2* mRNA level than those from the adult group (Fig. 2D; *P* < 0.001).

The *Pot1a* mRNA profile in the adult group exhibited low levels in the primary and secondary follicles, which progressively increased from the secondary to antral stages (Fig. 2E; *P* < 0.001). In the aged group, the low *Pot1a* mRNA levels observed in the primary and secondary follicles increased in the preantral follicles (*P* < 0.01), and significantly decreased in the antral follicles (*P* < 0.001). A comparison of the *Pot1a* profiles between the two groups revealed that although the primary and secondary follicles of the aged group possessed higher levels compared to their adult counterparts (*P* < 0.001), no difference was detected in the preantral follicles. Conversely, we observed a lower *Pot1a* mRNA level in the antral follicles of the aged group than in the same follicle type of the adult group (Fig. 2E; *P* < 0.001).

### The TERT protein levels in the follicles

**Figure 3:**
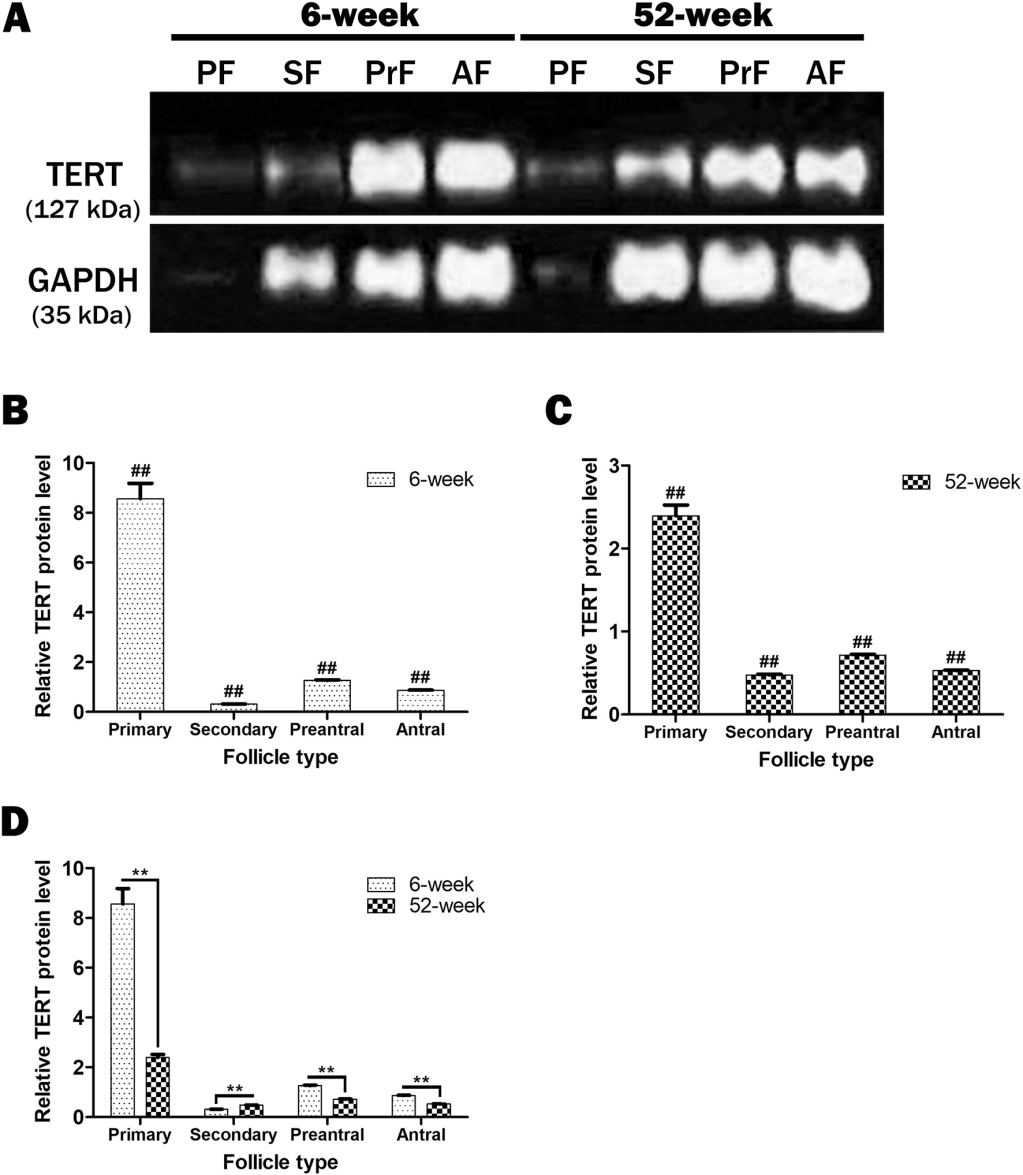
The TERT protein levels in the ovarian follicles from primary to antral stages, obtained from adult (6-week-old) and aged (52-week-old) groups. In this analysis, we used primary (n = 60 and 46), secondary (n = 77 and 58), preantral (n = 59 and 64), and antral (n = 56 and 38) follicles from 14 adult and 9 aged mice, respectively. (**A**) Western blotting (WB) results showing bands corresponding to the TERT and glyceraldehyde-3-phosphate dehydrogenase (GAPDH) proteins in the isolated follicles. GAPDH (in the molecular weight of 35 kDa) was used as an internal loading control. All the follicles either from adult or aged groups possessed the TERT protein (in the molecular weight of 127 kDa) at different levels. The bands produced via WB were analyzed using the ImageJ software program version 2.0 (bundled with 64-bit Java 1.8.0_172; NIH, Bethesda, Maryland, USA, URL link: https://imagej.nih.gov/ij/download), and the results were presented as a column chart. *PF* primary follicles, *SF* secondary follicles, *PreF* preantral follicles, *AF* antral follicles. (**B**) The relative TERT protein levels in the follicles from the adult group. The highest and lowest TERT protein levels were observed in the primary (*P* < 0.01) and secondary (*P* < 0.01) follicles, respectively. It should be noted that there were statistical significances among all follicle types (^##^*P* < 0.01). (**C**) The relative TERT protein levels in the follicles from the aged group. It progressively decreased from primary to antral follicles except for the secondary follicles, where a marked reduction was observed (*P* < 0.01). It is worth noting that there were statistical significances among all follicle types (^##^*P* < 0.01). (**D**) The comparison of relative TERT protein levels in the follicles between adult and aged groups. In the aged group, we detected lower TERT protein levels in the primary, preantral, and antral follicles (*P* < 0.01), but higher levels in the secondary follicles (*P* < 0.01) when compared to those of their adult counterparts. There were statistical significances among all follicle types (**P < 0.01). Here, the statistical significances in B and C were evaluated using one-way ANOVA test and the Student–Newman–Keuls post hoc test (denoted by ^##^); the student’s *t*-test (denoted by **) was used in D. The data obtained are presented as means ± standard deviations (SDs; arising from two technical repeats).

We further analyzed the TERT protein levels in the follicles obtained from the adult and aged groups at each of the four developmental stages. All the ovarian follicles exhibited different TERT protein levels (Fig. 3A and Supplementary file). An evaluation of the relative TERT protein levels using the ImageJ software program showed that in the adult group the highest level of TERT protein detected in the primary follicles significantly decreased in the secondary follicles (*P* < 0.01), increased in the preantral follicles (*P* < 0.01), and decreased in the antral follicles (*P* < 0.01; Fig. 3B). A similar distribution was observed in the aged group (Fig. 3C), where in the highest level of TERT protein detected in the primary follicles dramatically decreased in the secondary follicles (*P* < 0.01), increased in the preantral follicles (*P* < 0.01) and then reduced in the antral follicles (*P* < 0.01). A comparison of each follicle type between adult and aged groups (Fig. 3D) revealed that the primary, preantral, and antral follicles from the aged group had significantly lower TERT protein levels than their adult counterparts (*P* < 0.01). By contrast, the secondary follicles from the aged group exhibited higher level compared to those from the adult group (Fig. 3D; *P* < 0.01).

### Telomerase activity in the follicles

**Figure 4:**
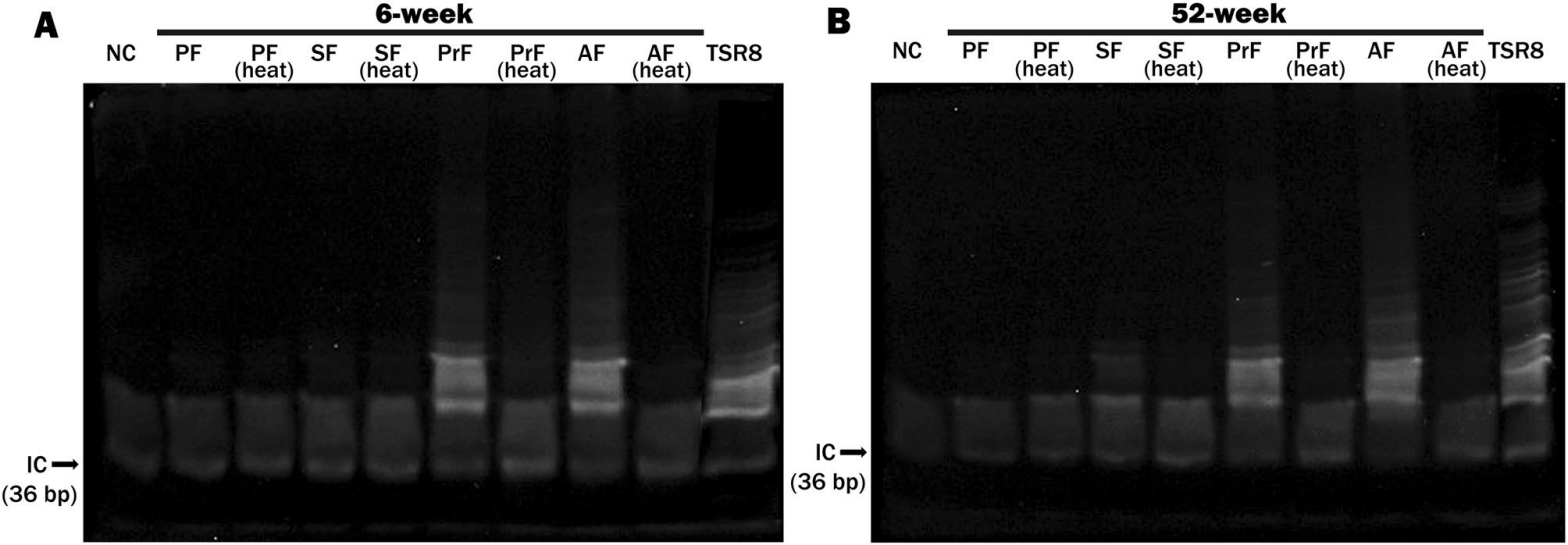
The telomerase activity in the ovarian follicles from primary to antral stages, obtained from adult (6-week-old) and aged (52-week-old) groups. For this analysis, we used primary (n = 34 and 21), secondary (n = 30 and 46), preantral (n = 30 and 25), and antral (n = 30 and 25) follicles collected from five adult and three aged mice, respectively. The TRAP assay results of the follicles from primary to antral stages in the adult (**A**) and aged (**B**) groups. As expected, internal control (IC) product was generated in all reaction tubes. An additional heat-treated control (abbreviated as heat) was run alongside each sample as telomerase is a heat-sensitive enzyme. In both adult and aged groups, we found that telomerase activity reached high levels in the preantral and antral follicles when compared to the primary and secondary follicles. It is worth noting that that this assay was technically repeated twice. *NC* negative control, *PF* primary follicles, *SF* secondary follicles, *PrF* preantral follicles, *AF* antral follicles, *TSR8* control template, *bp* base pair.

Telomerase activity in the follicles from the adult and aged groups was analyzed at each of the four developmental stages using the TRAP method (Fig. 4A, B, and Supplementary file). In the adult and aged groups, this activity sharply increased in the preantral and antral follicles when compared to the primary and secondary follicles. Importantly, the ovarian follicles from primary to antral stages of the adult and aged groups exhibited similar telomerase activity distribution during folliculogenesis.

## Discussion

Telomere shortening alongside reproductive aging is associated with the loss of fertility at the later stages of the female’s lifespan^6,33^. Age-associated meiotic defects that may derive from telomere attrition in the oocytes also lead to loss of female fertility^34,35^. The primary reasons for telomere shortening may be related to alteration of reproductive hormones (e.g., estrogen and progesterone) level, increased reactive oxygen species (ROS) profiles, and mitochondrial dysfunction. In the present study, we determined for the first time that the altered *Tert*, *Terc*, *Trf1*, *Trf2*, and *Pot1a* gene expression, TERT protein levels and telomerase activity in the aged ovarian follicles which may mediate shortening of telomeres and loss of fertility during biological aging^35,36^. These alterations might originate from changes in the reproductive hormones and enhanced ROS levels at the later periods of lifespan. Further research in this area is required to uncover any potential relationship that may exist.

To the best of our knowledge, a limited number of studies have been aimed to evaluate the expression patterns of the *Tert*, *Terc*, *Trf1*, *Trf2*, and *Pot1a* genes in the mammalian ovarian follicles. Here, we found that the primary, secondary, and antral follicles except for the preantral follicles exhibited similar *Tert* and *Terc* gene expression levels between adult and aged groups. This occurrence suggests that the basic telomerase components, *Tert* and *Terc*, may be regulated by common factors and remain unchanged during ovarian aging at these follicular stages. However, the discrepancy in the preantral follicles may have resulted from exposure to different factors or to different levels of these factors, which consequently affected gene expression. The expression of both *Tert* and *Terc* genes may be regulated by different factors during folliculogenesis, possibly in a gonadotropin-dependent manner. One such factor could be estradiol, whose level gradually decreases during aging^37,38^. In 2011, Bayne et al. documented that estradiol deficiency leads to a decreased *Tert* gene expression in the mouse ovaries^39^. Parallel to this study, an exogenous estradiol treatment to ovariectomized rats led to a significant increase of *Tert* mRNA levels in the heart, liver, and brain tissues^40^. Estradiol most likely modulates the *Tert* gene expression by binding to the estrogen response element (ESE) located on its promoter^41^. A recently published investigation reported that pregnant mare's serum gonadotropin [PMSG, 10 IU/mL; an FSH (follicle stimulating hormone) analog], enhanced TERT protein and mRNA levels as well as telomerase activity in the rat granulosa cells^42^. Overall, these findings suggest that the fluctuating *Tert* gene expression in the follicles during aging may arise from altered estradiol and FSH levels as well as from changes in their receptors.

We also observed that the relative levels of *Trf1*, *Trf2*, and *Pot1a* mRNAs in the antral follicles predominantly decreased in the aged group. As the late stages of folliculogenesis including preantral and antral follicles occur in a gonadotropin-dependent manner^43^, the decreased expression of these genes may be associated with altered gonadotropins and/or their receptor profiles during biological aging. Interestingly, the secondary follicles from the aged group displayed higher *Trf1*, *Trf2*, and *Pot1a* mRNA levels as well as increased *Tert* and *Terc* gene expression compared to those from the adult group. This surge may have originated from increased or decreased amounts of the factors that stimulate transcriptional activity of these genes in the secondary follicles from the aged group. In more detail, changed expression of several genes in the aged secondary follicles^44^ may lead to transcriptional stimulation via increasing transcriptional activators that might result in an increased expression of these telomere-related genes. Another possible reason of this increase may be arisen from enhanced FSH level in the aged mice^45^. As FSH is associated with increasing transcriptional activity^46,47^, the same effect might have occurred in the secondary follicles for these genes. Thus, as the mechanisms underlying the expressional control of telomere-related genes in mammalian follicles remain unknown, further studies are required to define those determinants.

Similar to our findings, Russo et al. reported that TERT protein was detected in the primary and preantral follicles (both in the granulosa cells and oocytes), and in the antral follicles (in the granulosa cells close to the antrum and cumulus cells, as well as in the oocytes) from pig ovaries^29^. Additionally, a correlation was established between telomere sizes and TERT protein levels in these follicles. Different from our finding, Lavranos et al. also measured the telomerase activity using PCR-ELISA method and observed a higher telomerase activity in the preantral follicles (60–100 µm in diameter) compared to that in small antral follicles (1 mm in diameter) from bovine ovaries^28^. This difference may arise from employing distinct methods, and the species being studied. Also, germinal vesicle (GV) oocytes in the human follicles at different developmental stages displayed higher telomerase activity than in the MII oocytes^48,49^. In rats, GV (from preantral and antral follicles) and MII oocytes also exhibited telomerase activity^27^. We revealed in the present study that telomerase activity levels sharply increased in the antral follicles in both adult and aged groups, possibly dependent on the granulosa cell content, and cytoplasmic and nuclear maturation status of the oocytes. The contrasting results regarding the TERT protein levels and telomerase activity in the follicles may be due to other potential roles of the TERT protein^50^ in the granulosa cells and oocytes during folliculogenesis.

In the present study, we determined that telomerase activity significantly increased in the preantral and antral follicles of the adult and aged groups compared to those of early follicles. As estradiol production increases in these follicular stages^51,52^, the increase of telomerase activity may derive from elevated estradiol levels. As previously described, estradiol leads to an increased *Tert* gene expression^39^, and expectedly estradiol deficiency can reduce telomerase activity and telomere length in the mouse ovaries. On the other hand, the telomerase activity distribution in the mouse ovarian follicles from primary to antral stages contradicts with the previous studies which have been comprehensively discussed in the review article published by our group^11^. We observed that telomerase activity gradually decreases from primordial to antral stages in the pig and bovine. Conversely, telomere length progressively increases in the same direction of follicular development. The differences between those studies and current work may result from differences related to studied species and used methodologies. Thus, further investigations are required in the same species using the similar methodologies in defining follicle types and measuring telomerase activity.

To the best of our knowledge, we have, for the first time, ascertained that TERT protein and mRNA levels, and telomerase activity exhibited different distributions in the ovarian follicles from primary to antral stages in the adult and aged groups. Specifically, although the primary follicles of both groups had highest TERT protein levels, their telomerase activity were at low levels. General consensus is that there is a close positive correlation among these parameters^53,54^, whereas it was displayed in a previous study that they did not show a parallel distribution during mouse brain development^55^. The same issue in the ovarian follicles indicates that there may be distinct regulation mechanisms for each parameter during folliculogenesis. Also, it is worth noting that the presence of dominant negative form of the TERT protein^56,57^ in the follicles, which does not contribute to telomerase activity, could affect the correlation status. Since there is no precise information regarding the specificity of the used TERT antibody whether recognizes only active TERT protein or its dominant-negative form or both forms, we do not rule out this issue without performing further molecular biological analyses. Thus, TERT protein and mRNA levels as well as telomerase activity should be separately evaluated to reach precise findings related to telomere biology.

Nevertheless, this study has some limitations. It is a descriptive study, in which a limited number of genes involved in telomere length regulation in the ovarian follicles were analyzed. Moreover, we only used *β-Actin* or *Gapdh* as a reference gene for normalizing the obtained data. As GAPDH can interact with the telomerase subunit *Terc* in cancer cells^58^, the concurrent use of additional reference genes in this type of studies would increase the reliability of the findings. Another methodological limitation was to perform all the technical analysis especially for TRAP assay in the pooled follicles without making biological repeats.

## Conclusion

In the current study, we determined significant changes in the relative expression levels of telomerase components (*Tert* and *Terc*), *Trf1*, *Trf2*, and *Pot1*a genes as well as TERT protein levels and telomerase activity in the ovarian follicles obtained from adult and aged mice. The decreased expression of these genes and TERT protein levels in the follicles of the aged mice may be associated with ovarian aging and loss of fertility appearing at the later stages of lifespan. Further investigations are required to characterize the intracellular signaling pathways responsible for inducing these changes in the ovarian follicles as well as in the oocytes and granulosa cells.

## Supplementary Information

Supplementary Information.
