# Supplementary material for: Telomere associated gene expression as well as TERT protein level and telomerase activity are altered in the ovarian follicles of aged mice
Source: Sci Rep. 2021 Jul 30;11:15569. doi: 10.1038/s41598-021-95239-5 (PMC8324818; doi:10.1038/s41598-021-95239-5)

**Telomere associated gene expression as well as TERT protein level and telomerase activity alter in the ovarian follicles of aged mice**

Esra Gozde Kosebent and Saffet Ozturk*****

Department of Histology and Embryology, Akdeniz University School of Medicine, Antalya, Turkey.

**Supplemental data**

**Supplemental data Figure 3.** The TERT levels in the ovarian follicles from primary to antral stages in the adult and aged groups. In this analysis, we used primary (n = 60 and n = 46), secondary (n = 77 and n = 58 ), preantral (n = 59 and n = 64), and antral (n = 56 and n = 38) obtained from 14 adult and 9 aged mice, respectively. The representative bands from left to right show marker, primary, secondary, preantral, and antral follicles of the adult group, and primary, secondary, preantral, and antral follicles of the aged group, respectively. Notably, the far away band at the right shows the TERT level in the mouse ovary tissue, which is used as a positive control.


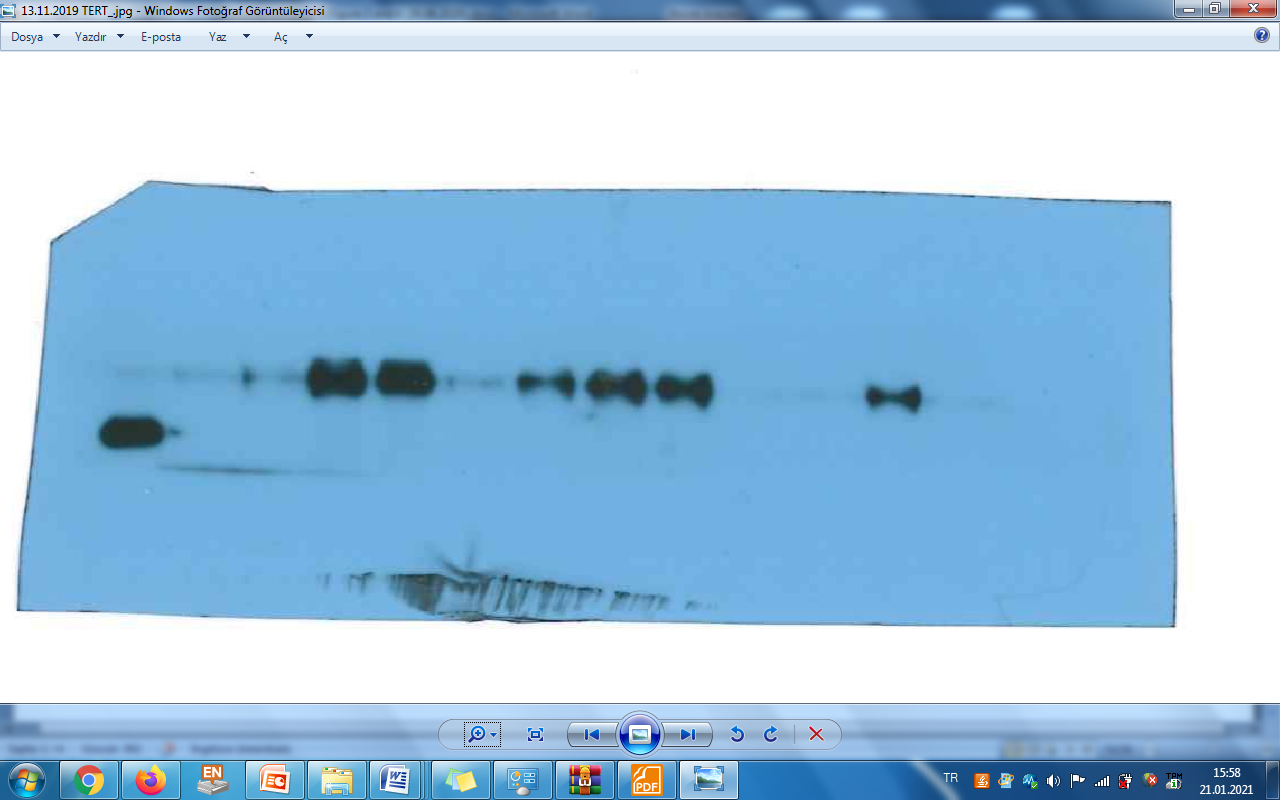


**Supplemental data Figure 3.** The GAPDH levels in the ovarian follicles from primary to antral stages in the adult and aged groups. In this analysis, we used (n = 60 and n = 46), secondary (n = 77 and n = 58), preantral (n = 59 and n = 64), antral (n = 56 and n = 38) obtained from 14 adult and 9 aged mice, respectively. The representative bands from left to right show marker, primary, secondary, preantral, and antral follicles of the adult group, and primary, secondary, preantral, and antral follicles of the aged group, respectively.


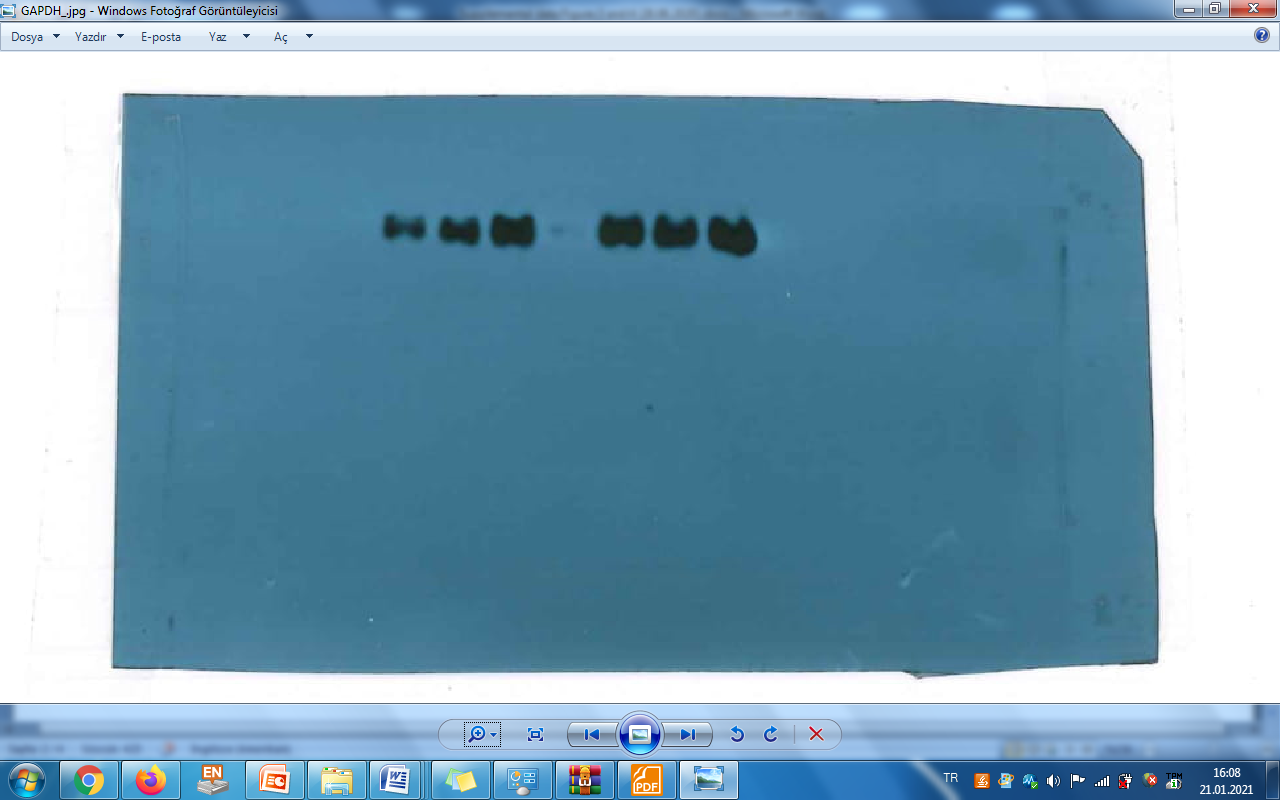


**Supplemental data Figure 4.** The telomerase activity in the ovarian follicles from primary to antral stages in the adult group. For this analysis, we used primary (n = 34), secondary (n = 30), preantral (n = 30 ), antral (n = 30) from 5 adult mice. The representative PCR bands from left to right show NC (negative control), primary, primary (heat), secondary, secondary (heat), preantral, preantral (heat), antral, antral (heat) follicles, and TSR8 template.


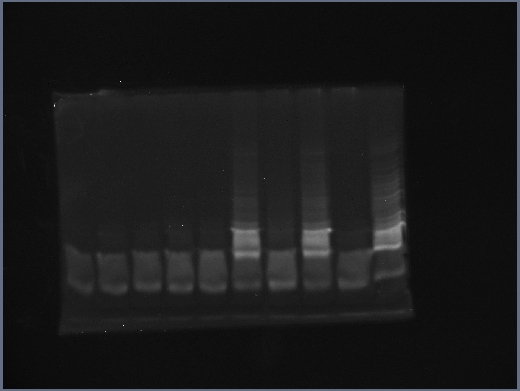


**Supplemental data Figure 4.** The telomerase activity in the ovarian follicles from primary to antral stages in the aged group. For this analysis, we used primary (n = 21), secondary (n = 46), preantral (n = 25), antral (n = 25) from 3 aged mice. The representative PCR bands from left to right show NC (negative control), primary, primary (heat), secondary, secondary (heat), preantral, preantral (heat), antral, antral (heat) follicles, and TSR8 template.


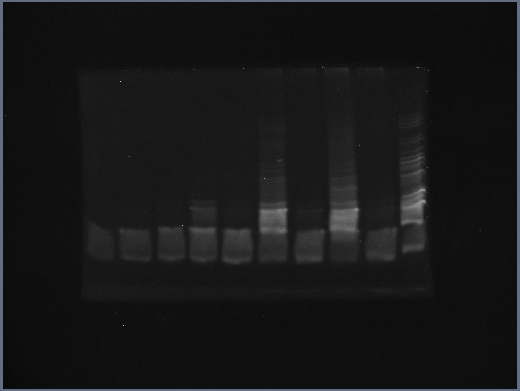

Supplement: Supplementary file 1 — Supplementary Information. [file 41598_2021_95239_MOESM1_ESM.docx]
